# Advanced Tracers in PET Imaging of Cardiovascular Disease

**DOI:** 10.1155/2014/504532

**Published:** 2014-10-15

**Authors:** Yesen Li, Wei Zhang, Hua Wu, Gang Liu

**Affiliations:** ^1^Department of Nuclear Medicine, The First Affiliated Hospital of Xiamen University, Xiamen 361003, China; ^2^Center for Molecular Imaging and Translational Medicine, School of Public Health, Xiamen University, Xiamen 361102, China; ^3^Department of Orthopedics, Sichuan Academy of Medical Sciences & Sichuan Provincial People's Hospital, Chengdu 610072, China; ^4^Sichuan Key Laboratory of Medical Imaging, North Sichuan Medical College, Nanchong 637007, China

## Abstract

Cardiovascular disease is the leading cause of death worldwide. Molecular imaging with targeted tracers by positron emission tomography (PET) allows for the noninvasive detection and characterization of biological changes at the molecular level, leading to earlier disease detection, objective monitoring of therapies, and better prognostication of cardiovascular diseases progression. Here we review, the current role of PET in cardiovascular disease, with emphasize on tracers developed for PET imaging of cardiovascular diseases.

## 1. Introduction

Cardiovascular diseases are progressive and there are a number of physiological and morphological changes that occur with aging that could alter cardiovascular function and subsequently increase risk of cardiovascular disease [1]. Despite considerable advances in prevention and treatment over the last decades, cardiovascular diseases remain the most frequent causes of death worldwide and represent a great challenge for modern research and medicine. To address this requires urgent development of sensitive and noninvasive methods for early detection and personalized treatment of cardiovascular diseases [2].

Traditional medical imaging techniques have been routinely used to provide early diagnoses and prognosis of cardiovascular diseases [3]. Ideally it would be possible to detect molecular and cellular processes early and characterize cardiovascular diseases before manifestation of gross anatomical features or physiological consequences. Current advanced molecular imaging modalities include positron emission tomography (PET), single photon emission computed tomography (SPECT), magnetic resonance imaging (MRI), and optical imaging, all of which could provide critical molecular and cellular level information for early diagnostics, advanced therapeutics, and better understanding of fundamental biological processes of cardiovascular disease [4–6].

Nuclear cardiac imaging has been indisputably the most reliable and accurate technique for noninvasive function characterization of coronary artery disease (CAD) [7]. Due to its clinical success in oncology, the availability of PET systems has increased dramatically since it was developed in 1975 [8], and as such has been able to contribute to the growth in cardiac imaging [9]. Generally, PET imaging with appropriate tracers is considered to have superior diagnostic accuracy for the detection of cardiovascular diseases [10, 11]. For example, myocardial perfusion imaging (MPI) with PET has superior diagnostic accuracy and safety profile, and it provides powerful and incremental risk stratification for patients with suspected or known CAD [12]. Furthermore, PET imaging with [^18^F]-fluorodeoxyglucose ([^18^F]FDG) has been the gold standard for myocardial metabolism assessment [13].

Recent advances in radiopharmaceuticals, hardware, and software of cardiac PET imaging have improved the diagnostic accuracy and risk assessment of patients with suspected cardiac disorders [14–17]. For example, the rapid evolution of hybrid PET/CT and PET/MR imaging with the advanced tracers has provided a new perspective on cardiac imaging by providing combined anatomic and functional evaluation of coronary disease and alterations in cardiac function. Because of the pivotal role of tracers in the realization of the power of cardiac PET imaging, the design and development of tracers is becoming one of the major subjects of cardiac PET imaging [18]. In this paper, we review the present state and future of cardiac PET imaging, with a focus on PET tracers and their utility in cardiovascular diseases.

## 2. PET: State-of-the-Art Modality for Cardiac Diagnostics

PET is advantageous among nuclear medicine imaging modalities, because some positron emitting isotopes (e.g., C, N, and O) are the principal constituents of bioorganic molecules and can be labeled to the bioorganic molecules without change to their physicochemical and biochemical properties. The most widely used radionuclide ^18^F is generally not a constituent of biomolecules but a bioisostere of a hydrogen or hydroxyl group [10].  Table 1 shows the PET radionuclides commonly used in cardiovascular disease imaging, which are often produced by cyclotron, except for ^82^Rb and ^68^Ga.

SPECT myocardial perfusion imaging is considered a reliable and widely available tool for use by physicians in cases of cardiovascular disease. However, recent studies have shown that PET offers increased accuracy and improved sensitivity compared to SPECT [19]. Indeed, several PET myocardial perfusion tracers (e.g., [^82^Rb]RbCl, [^13^N]NH_3_, and [^15^O]H_2_O) used for assessment of myocardial perfusion have produced higher quality images than technetium SPECT imaging [20].

In addition, PET has several technical advantages over SPECT: (1) most importantly PET has 2-3 orders of magnitude higher sensitivity than SPECT and the spatial resolution of PET can reach 4 mm compared to 10–12 mm in whole-body SPECT scans, or 6-7 mm in new dedicated cardiac SPECT equipment (i.e., CZT cameras) [21, 22]; (2) PET has much higher temporal resolution than SPECT [19], with a hybrid PET/CT system providing accurate depth-independent attenuation correction [23]; (3) PET tracers have a shorter physical half-life and higher extraction fractions compared to SPECT tracers, leading to less radiation burden to patients and superior image quality in comparison to standard SPECT [24, 25], particularly for cases where image quality can be degraded with SPECT, such as in obese patients or in women where a breast attenuation artifact could lead to a false positive result [24]. Furthermore, the use of 3D acquisitions represents an important milestone in myocardial perfusion PET imaging and could significantly shorten imaging protocols, reduce radiation exposure, and increase its use in PET cardiac imaging [26, 27].

## 3. MPI Tracers

MPI is a noninvasive test that involves injection of a small amount of radioactive tracer into the body to depict the distribution of  blood flow to the heart. MPI is used to identify areas of reduced blood flow (perfusion) to the heart muscle which may indicate CAD. Due to its high sensitivity and temporal resolution MPI with PET is considered the gold standard for quantifying myocardial perfusion (mL/min/g), both at rest and during stress [24].

To achieve high quality imaging, an ideal PET myocardial perfusion tracer (1) should have a high first-pass extraction fraction from blood to tissue and retention in the heart, while allowing a low clearance from tissue to blood at baseline to high flow rates; (2) the myocardial tracer uptake should correlate with the blood flow rate; (3) should have minimal uptake and retention in the lungs and liver to avoid interference with myocardial imaging; with long retention in cardiac tissue and* in vivo *stability; (4) could be synthesized efficiently, reliably, and with full automation; (5) should have a relatively long half-life and be possible to distribute to institutes without an on-site cyclotron [2]. However, many of the PET myocardial perfusion tracers are unfavorable for MPI.

**Figure 1:**
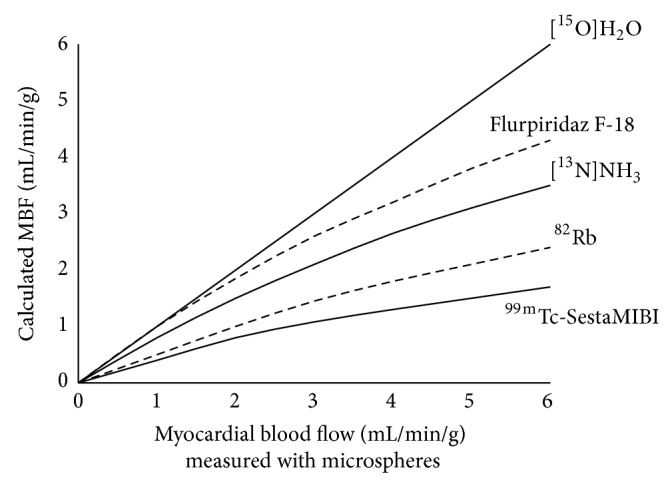
Correlation between the calculated myocardial blood flow (MBF) base imaging studies with myocardial perfusion tracers and the expected MBF [2, 122].

The currently used PET myocardial perfusion tracers are [^15^O]H_2_O, [^13^N]NH_3_, and [^82^Rb]RbCl. [^15^O]H_2_O is metabolically inert and can diffuse freely across the capillary and cellular membranes, leading to the ideal first-pass extraction fraction as shown in Figure 1. Thus, the net extraction correlates linearly with blood flow [29, 30], which is considered ideal for measuring myocardial blood flow [31]. In addition, the short physical half-life of [^15^O]H_2_O could afford multiple flow measurements at short intervals. However, a comparison study of [^15^O]H_2_O versus [^13^N]NH_3_ for quantification of myocardial blood flow in humans found that [^15^O]H_2_O yielded more heterogeneous estimates of regional myocardial blood flow than [^13^N]NH_3_ [32] and that because of its freely diffusible nature [^15^O]H_2_O is not retained in the myocardium. Therefore, [^15^O]H_2_O uptake between the myocardium and blood pool reach equilibrium rapidly, making it impossible to distinguish ventricular from myocardial activity and thereby precludes generating appropriate images of myocardial perfusion. [^15^O]CO, which permits labeling of the vascular volume, has been used in combination with [^15^O]H_2_O for correction of the high ^15^O activity in the blood pool [33]. However, mathematical models could be used to produce parametric imaging of perfusion without additional [^15^O]CO blood pool scan sequences [34, 35]. Additionally, due to its short half-life [^15^O]H_2_O requires an on-site cyclotron. Although these limits have hampered the use of [^15^O]H_2_O for PET perfusion imaging in routine clinical practice, due to its excellent radiokinetic properties, [^15^O]H_2_O has gained wide popularity for research purposes [36, 37].

^82^Rb is a generator product with a physical half-life of  76 s, which acts as a potassium analogue and is taken up by the energy-dependent Na/K-ATPase and extracted similarly to thallium-201 and lower to ^13^N-ammonia and ^15^O-water [24]. Studies have demonstrated that PET MPI with ^82^Rb performs superior to SPECT in sensitivity, image quality, interpretive certainty, and diagnostic accuracy [38, 39]. With a single rest and pharmacologic stress ^82^Rb PET/CT scanning, Kim et al. have demonstrated that the combination of MPI, coronary artery calcium (CAC), coronary flow reserve (CFR), and thoracic aorta calcium (TAC) has complementary roles in intermediate risk patients [40]. Due to its distinct advantage of not requiring an on-site cyclotron, ^82^Rb is the most frequently used clinical myocardial perfusion PET tracer, and the clinical application of ^82^Rb and PET for myocardial perfusion imaging is expected to grow. The acquisition time of PET/CT protocols with ^82^Rb requires only 25–30 min, compared to at least 2.5 h and as much as 4 h, for SPECT strategies [41]. Moreover, the radiation exposure from cardiac imaging procedures is reasonably low due to its ultrashort physical half-life [42].

However, there are some limitations associated with the use of  ^82^Rb. Firstly, ^82^Rb has the lowest myocardial extraction fraction (65% of first-pass extraction) among the currently available PET perfusion tracers, and its nonlinear extraction at higher flow leads to underestimation of MBF [43, 44]. Secondly, the maximum kinetic energy of ^82^Rb is higher than the other radionuclides shown in Table 1; therefore, the tissue positron range of ^82^Rb is longer, resulting in a relatively low spatial resolution [36]. Additionally ultrashort half-life of  ^82^Rb can result in oversaturation at the beginning of the acquisition, as well as low count statistics and imaging noise in later phases [2, 44]. For these reasons, when using ^82^Rb patients are usually stressed pharmacologically, rather than by exercise [43], while the mode of ^82^Rb myocardial uptake may also change when cellular metabolism is altered [45]. These factors make ^82^Rb less than ideal for absolute quantification of myocardial blood flow.

The FDA approved [^13^N]NH_3_ for myocardial perfusion imaging in 2000. Although NH_3_ is a freely permeable to cell membranes and NH^4+^ is a relatively impermeable cation to cell membranes, the mechanism of cellular localization of [^13^N]NH_3_ is not yet fully understood. Diffusing into the cellular membrane followed by trapping by glutamine, glutamic acid, and carbamyl phosphate, is one of the described mechanisms of [^13^N]NH_3_ cellular localization [46]. Due to its high first-pass extraction fraction (>80%) and linear myocardial uptake over a wide range of MBF, [^13^N]NH_3_ is a more practical standard perfusion tracer compared to other tracers [43, 47, 48]. The longer physical half-life (9.8 min) of [^13^N]NH_3_ compared to [^15^O]H_2_O leads to prolonged retention in myocardium, preferential distribution into the myocardium, and ultimately better image quality. As [^13^N]NH_3_ and [^15^O]H_2_O perform similarly in the estimation of myocardial blood flow in humans [32], an increasing number of publications have demonstrated its substantial diagnostic and prognostic value [49–52]. However, [^13^N]NH_3_ myocardial uptake depends not only on perfusion but also myocardial cell viability, metabolic conditions, and the integrity of the cellular membrane, limiting its value as a myocardial perfusion tracer [53].

**Figure 2:**
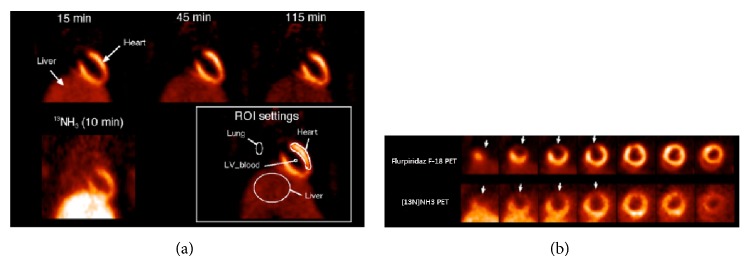
(a) Flurpiridaz F-18 PET images of healthy rat and [^13^N]NH_3_ PET image in coronal view. (b) Images of rat with permanent left coronary artery occlusion using Flurpiridaz F-18 PET and [^13^N]NH_3_ PET. Arrows indicate localization of myocardial infarction (from [58] with permission).

Flurpiridaz F-18 (^18^F-BMS-747158-02) is a derivative of the pyridazinone insecticide pyridaben, which is known to bind tightly to mitochondrial complex I, with high affinity in myocardial tissue [54, 55]. The high first-pass extraction fraction (94%) of flurpiridaz F-18 [56] has several advantages, including detection of milder perfusion defects, and better accuracy in reflecting the true extent of perfusion defects compared to ^201^Tl, the ^99m^Tc-labelled tracers, or ^82^Rb. Flurpiridaz F-18 also offers better image quality and higher diagnostic certainty, compared to Tc-99m SPECT [57]. Additionally, compared with [^13^N]NH_3_, flurpiridaz F-18 was shown to produce improved contrast and higher resolution, resulting in better delineation of induced perfusion defects with lower injected dose of tracers (Figure 2) [58]. In a phase 1 study using flurpiridaz F-18 no drug-related adverse events were identified, and it was well tolerated in all subjects [59]. A phase II study using flurpiridaz F-18 has proved that PET MPI with flurpiridaz F-18 has a higher sensitivity than SPECT, with an increase in diagnostic certainty for PET versus SPECT [60]. As such, flurpiridaz F-18 might be close to an ideal myocardial perfusion tracer and is currently being evaluated in phase III clinical studies. Exercise stress testing offers important clinical and hemodynamic information in addition to myocardial perfusion. Flurpiridaz F-18 can potentially be used in exercise stress testing by injection of tracer during peak exercise and postinjection imaging because of the longer half-life of ^18^F. However, the long physical half-life of flurpiridaz F-18 may limit its use in protocols involving repeated measurements on a single day; therefore, protocols involving stress and rest studies should perform on separate days or protocols involving repeated measurements on a single day should correct for the residual activity of the first acquisition and the validity of a tracer reinjection protocol.

Several other ^18^F-labeled perfusion PET tracers have been introduced for the evaluation of myocardial perfusion. Yu et al. [61] found that another mitochondrial complex I inhibitor, ^18^F-RP1004, has a higher initial liver uptake compared to flurpiridaz F-18 (^18^F-BMS-747158-02), possibly due to the higher lipophilicity of ^18^F-RP1004. Mou et al. [62] synthesized [^18^F]FP2OP, and biodistribution studies in mice have shown that [^18^F]FP2OP has a significant high heart uptake, as well as good heart/liver, heart/lung, and heart/blood ratios and has therefore been proposed as a potential new MPI agent for PET. ^18^F-FTPP is an analog of tetraphenylphosphonium (TPP^+^) cation that concentrates in mitochondria, also demonstrated promising characteristics as a PET MPI tracer. Biodistribution and imaging studies with ^18^F-FTPP in rats have shown rapid accumulation of activity in the heart (1-2 min) with stable retention for at least 1 h. Heart uptake of ^18^F-FTPP in occluded heart ROIs was comparable to that of [^13^N]NH_3_ in rabbits [63, 64]. In addition, some coordination compounds of metal radionuclides have been developed for myocardial imaging, such as ^62^Cu-PTSM [65] and ^68^Ga-BAPEN [63, 64].

## 4. Myocardial Metabolism Imaging 

PET MPI is important for providing insight into substrate “feed” to the heart, while PET imaging of heart metabolism will lead to a deep understanding of the biochemical pathways of the heart and their disease behaviors. Despite rapid growth in our understanding of the relationship between altered myocardial metabolism and cardiac disease, there remains much to learn. There is a growing demand for accurate noninvasive imaging approaches of myocardial substrate metabolism, with one of the most notable application being the assessment of myocardial viability. Myocardial viability studies are crucial for differentiation of patients who can benefit from revascularization and those without myocardial viability, for whom there is no apparent benefit from revascularization over medical therapy.

Myocardial viability refers to dysfunctional myocardium due to ischaemia, but with the potential to recover its function. In patients with ischaemic left ventricular (LV) dysfunction, those with viable myocardium should be considered for revascularization rather than cardiac transplantation, while patients with nonviable myocardium will not benefit from high-risk percutaneous coronary interventions or surgery [66]. There are two pathophysiological phenomena currently used to describe the viable myocardium: myocardial stunning and hibernation. Myocardial stunning refers to the phenomenon of contractile dysfunction of the ischemic heart which persists for hours or weeks even after reperfusion, in which the recovery of myocardial function depends on the duration and severity of ischemia as well as rate of coronary blood flow. Myocardial hibernation refers to dysfunctional myocardium, which is in a state of metabolic downregulation resulting from sustained hypoperfusion. In these cases viable myocardium may take weeks or months to recover once flow is restored. One of the key differences between these two concepts is that resting myocardial perfusion is normal/near normal in stunning but is reduced in hibernation. Both conditions are reversible and hibernation could be considered as the summation of repetitive and cumulative stunning; they may contribute to LV dyssynchrony and heart failure in patients with CAD [67]. Currently, PET is regarded as the gold standard for viability assessment.

Generally, cardiac contraction requires energy generated by aerobic metabolic pathways; therefore, measurements of myocardial oxygen consumption would provide direct assessments of the myocardial oxidative metabolism. Noninvasive quantitative measurements of myocardial oxygen consumption in human can be made by PET imaging with [^15^O]O_2_ inhalation. Iida et al. [68] described a kinetic model for quantification of regional myocardial oxygen consumption and oxygen extraction fraction and it is the first approach to allow direct quantitative determination of oxygen extraction fraction and oxygen metabolism to be made noninvasively on a regional basis. Yamamoto et al. [69] validated the method for noninvasive quantification of regional myocardial oxygen consumption and oxygen extraction fraction by PET with [^15^O]O_2_ inhalation using three distinct analytical approaches, each of which provides accurate measurements of regional myocardial oxygen consumption and oxygen extraction fraction compared with invasive reference techniques. Major disadvantages of [^15^O]O_2_ include the need for multiple tracers to calculate myocardial blood flow and blood volume and, due to its short physical half-life, requirement for an on-site cyclotron.

Acetate is a 2-carbon fatty acid (FA) that is rapidly picked-up by cells and metabolized into acetyl-CoA, which enters the tricarboxylic acid cycle. [^11^C]acetate has been proposed as a tracer of oxidative metabolism, with [^11^C]acetyl-CoA being rapidly converted into CO_2_ and water [70]. Brown et al. [71] were first to apply [^11^C]acetate to the study myocardial oxygen utilization in New Zealand rabbits and demonstrated that [^11^C]acetate has a high extraction fraction and rapid decline of blood pool radioactivity* in vivo*. Brown et al. [72] further demonstrated that the efflux of labeled CO_2_ after the administration of [^11^C]acetate reflects myocardial oxygen consumption (MVO_2_) and that it is only minimally influenced by the changes in substrate utilization (<4%). The initial uptake of [^11^C]acetate is dependent on myocardial blood flow and has a relatively high first-pass extraction in myocardial tissue. It has also been shown that [^11^C]acetate can be used to quantify MBF with good agreement to actual MBF [73]. In addition, [^11^C]acetate PET has been shown to have high sensitivity for detection of recurrent prostate cancer and metastases [74]. However, [^11^C]acetate availability is limited by the short half-life of ^11^C which must be produced on-site.

The myocardium has a high rate of energy consumption for contractile function and relies on a variety of metabolic substrates, with most of the energy required for contraction coming from oxidation of FAs under normal conditions. However, there is a decrease in FA oxidation and increased glycolysis and glucose oxidation under conditions of ischemia [75]. Indeed, decreased FAs use and enhanced glucose use is the metabolic signature of the ischemic heart, and therefore PET imaging of myocardial metabolism focuses on FAs and glucose [76].

FAs are rapidly and avidly extracted by the heart, but uptake and subsequent metabolism are complex. Once activated to acyl-CoA, FAs can be degraded by *β*-oxidation in mitochondria. The transfer of acyl residues into the mitochondrial matrix relies on the carnitine-dependent transport system, which serves as the primary regulatory site for FA oxidation [77]. Ischemia results in insignificant loss of carnitine from the myocardium and accumulation of long-chain acyl-carnitine and acyl-CoA [78]. [^11^C]palmitate, one of the earliest tracers developed, has been used extensively in imaging of the heart and can be used to evaluate the enzymatic activity of carnitine-palmitoyl transferase I [79]. Early studies have shown that the size of the defect identified by PET imaging after [^11^C]palmitate administration enabled quantification and localization of myocardial infarcts in patients [80]. There are large zones of intensely depressed accumulation of [^11^C]palmitate in patients with ischemic cardiomyopathy, suggesting that cardiomyopathic states are associated with alteration in FA metabolism [81]. However, the clinical use of [^11^C]palmitate has been limited by its complex kinetics and backdiffusion of the unmetabolized tracer from the myocytes, as well as its relatively complicated synthesis and need for an on-site cyclotron.

**Figure 3:**
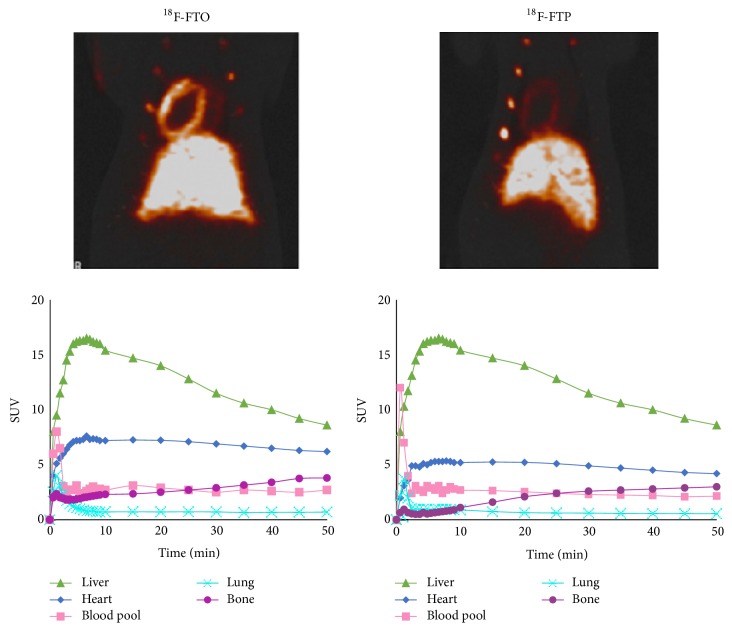
Early time-activity curves for ^18^F-FTO and ^18^F-FTP in rat heart (left ventricle), liver, lung, bone (rib), and blood pool and PET thoracic images acquired at 55–115 min after administration (from [86] with permission).

FA analogues act as false substrates or inhibitors of FA metabolism, and many PET FA analogue tracers have been designed to reflect myocardial *β*-oxidation. 14-(R,S)-^18^F-fluoro-6-thia-heptadecanoic acid ([^18^F]FTHA) is a long-chain fatty acid analogue with a high first-pass uptake rate, which becomes trapped in the mitochondria after incomplete *β*-oxidation. Inhibition of FA oxidation resulted in 81–87% decrease in uptake of [^18^F]FTHA in rat heart, suggesting that accumulation of [^18^F]FTHA in the myocardium may be useful in assessment of the *β*-oxidation rate of  long-chain FAs [82]. Although there is an increased myocardial FA uptake of [^18^F]FTHA in patients with heart failure [83], studies have shown [^18^F]FTHA to be insensitive to the inhibition of *β*-oxidation by hypoxia [84]. 16-[^18^F]fluoro-4-thia-palmitate ([^18^F]FTP) was developed to improve sensitivity to the FA oxidation by hypoxia and was found to be capable of detecting the influence of exogenous long-chain FAs on myocardial oxidation rates. However, myocardial retention of [^18^F]FTP was suboptimal [85]. Furthermore, ^18^F-fluoro-4-thia-oleate ([^18^F]FTO) has been shown to have a higher myocardial retention and superior myocardial imaging compared to [^18^F]FTP, as well as having many properties of a promising metabolically trapped FA oxidation tracer (Figure 3) [86].

FAs play a major role in the metabolism of the heart; however, glucose becomes the major substrate for the myocardium under ischemic conditions [87, 88].

^18^F-fluorodeoxyglucose [^18^F]FDG is an analogue of glucose and is used to visualize glucose metabolism* in vivo*. [^18^F]FDG is currently the most widely used PET tracer worldwide, particularly in oncology. [^18^F]FDG is taken up by myocytes via facilitated diffusion through a sarcolemmal glucose transporter, followed by phosphorylation by the hexokinase to [^18^F]FDG-6-phosphate. After phosphorylation, the molecule is intracellularly trapped without undergoing further metabolism. As a result, [^18^F]FDG is effectively fixed in the myocardium in a manner that is proportional to the transport rate of glucose transporter and the activity of hexokinase. In cardiology, [^18^F]FDG has been approved for viability assessment with PET by FDA and can be considered the gold standard for detection of the reversibility of injured myocardium in the clinical setting [89].

Viable myocardium has a preserved or increased glucose utilization in hypoperfused and dysfunctional myocardium (flow-metabolism mismatch), whereas nonviable segments have a concordant reduction in both blood flow and FDG uptake (matched defect) [9]. In the fasting state, [^18^F]FDG myocardial uptake is low and heterogeneous. To maximize myocardial uptake, [^18^F]FDG is normally administrated after an oral glucose load or insulin clamp. Acipimox, which decreases serum FAs by inhibiting lipolysis and indirectly stimulates cardiac [^18^F]FDG uptake, is an alternative clamp option. This approach is able to produce good image quality in both clinic and research settings [90, 91]. The first-pass extraction fraction of [^18^F]FDG is not only high but also adequate for most studies. Based on regional uptake of [^18^F]FDG, images are usually interpreted semiquantitatively. Knuuti et al. [92] found that absolute quantification of regional myocardial glucose utilization does not enhance the diagnostic accuracy of [^18^F]FDG PET for viable myocardium, likely due to larger variability of regional myocardial glucose utilization. In clinical practice, viable myocardium studies with [^18^F]FDG are usually combined with myocardial perfusion with [^13^N]NH_3_ or ^82^Rb. Cardiovascular metabolic imaging with [^18^F]FDG not only is limited to the assessment of myocardial viability, but also can be used to assess the severity of inflammation in carotid plaques [93].

## 5. Other Biomarker-Based Probes

Angiogenesis is the development of new capillaries from existing microvessels. Myocardial ischemia and infarctions result in hypoxia which stimulates angiogenesis [94]. Many factors have been found to contribute to the process of angiogenesis, and vascular endothelial growth factor (VEGF) is considered to be the most predominant factor [95]. In addition, integrins also participate in a number of processes related to angiogenesis [96], with *α*_*v*_ *β*_3_ being the most abundant integrin expressed on the surface of proliferating endothelial cells [97]. VEGF and *α*_*v*_ *β*_3_ integrin have been identified as favorable biomarkers for imaging angiogenesis using PET tracers for these targets [98, 99].

Many PET tracers have been developed for imaging of VEGF receptors in myocardial angiogenesis [100]. ^64^Cu-DOTA-VEGF121 was developed for imaging VEGF receptor in a rat myocardial infarction model and provides a useful tool for study of CAD biology [101]. PET/CT with reporter gene technology has been used to identify expression of VEGF_121_ in myocardium of healthy pigs after regional adenoviral transfer of the VEFG_121_ gene, with results suggesting that this technology could lead to targeted therapeutic interventions with an imaging modality for assessment of the clinical usefulness of therapy [102].

**Figure 4:**
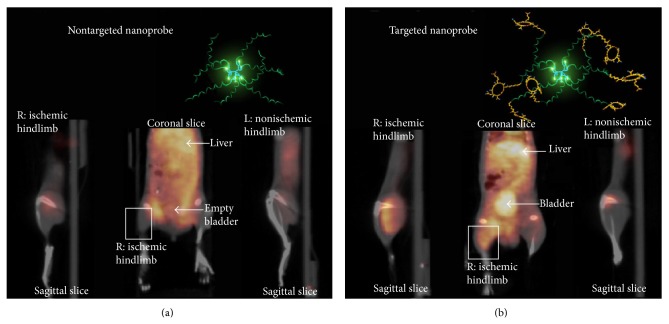
Noninvasive PET/CT images of angiogenesis induced by hindlimb ischemia in a murine model. Uptake of targeted dendritic nanoprobes was higher in ischemic hindlimb (a) as compared with control hindlimb (b) (from [106] with permission).

There has been significant focus on imaging of the *α*_*v*_ *β*_3_ integrin with the RGD (Arg-Gly-Asp) motif. The RGD peptides, which were identified by* in vivo* screening of phage-display peptide libraries, have high affinities for the *α*_*v*_ *β*_3_ integrin. PET with ^18^F-AlF-NOTA-PRGD2 allows noninvasive visualization of ischemia/reperfusion-induced myocardial angiogenesis longitudinally, and its favorable pharmacokinetics and easy production should facilitate its future clinical translation for lesion evaluation and therapy response monitoring in patients with occlusive cardiovascular diseases [103]. [^18^F]Galacto-RGD allows quantification of *α*_*v*_ *β*_3_ expression* in vivo* and has been successfully used to image angiogenesis in a patient 2 weeks after myocardial infarction [104, 105]. A biodegradable dendritic PET imaging nanoprobe developed by Almutairi et al. [106] has been shown to have enhanced bioavailability,* in vivo* radiostability, and increased binding specificity to *α*_*v*_ *β*_3_ integrin (Figure 4). In addition, this nanoprobe may allow for targeted drug delivery, potentially providing an approach for translation of novel theranostic tracers into clinical practice.

The *β*-adrenoceptor (*β*-AR) plays an important role in heart failure, with low *β*-AR density a direct reflection of decreased contractility in the heart, an important hallmark of heart failure. Although, clinical studies have demonstrated heart failure, patients could benefit from *β*-blocker therapy [107], it remains difficult to predict whether heart failure patients will respond favorably to *β*-blockers. De Jong et al. [108] have demonstrated that (S)-[^11^C]CGP12388 can be used to directly measure myocardial *β*-AR density in patients, revealing a significant reduction of *β*-AR density in patients with idiopathic dilated cardiomyopathy. While Naya et al. [109] have demonstrated that PET with ^11^C-CGP12177 can predict the improvement of cardiac function in patients with idiopathic dilated cardiomyopathy (IDC) after long-term carvedilol treatment.

Sympathetic nerves play key roles in cardiac physiology and aging-related cardiovascular diseases. Most of the norepinephrine in the human heart is removed by neuronal uptake [110] and studies have suggested that decreased neuronal uptake activity may contribute to congestive heart failure [111]. 6-[^18^F]fluopamine is an analogue of dopamine that has a similar metabolic fate to that of endogenous catecholamines. 6-[^18^F]fluopamine not only could assess sympathetic innervation and function in the heart, but can also detect altered cardiac uptake of catecholamines activity [112, 113].

Sympathetic nerves are exquisitely sensitive to ischemia [114], and regional cardiac sympathetic nerve dysfunction might have a role in its association with sudden cardiac death. ^11^C-hydroxyephedrine ([^11^C]HED) is a norepinephrine analogue that can be used for the noninvasive evaluation of neuronal integrity using PET. Studies have demonstrated profound reductions in regional uptake of norepinephrine by sympathetic nerves with [^11^C]HED imaging in a porcine model of  hibernating myocardium [115]. However, PET imaging with [^11^C]HED cannot accurately quantify regional nerve densities due to the rapid neuronal uptake rates. Raffel et al. [116] recently synthesized N-^11^C-guanyl-(-)-meta-octopamine ([^11^C]GMO), which has a much slower NET transport rate and is trapped in storage vesicles. Initial results have shown that [^11^C]GMO provides robust and sensitive quantitative measurements of regional cardiac sympathetic nerve density and might be able to detect mild-to-moderate sympathetic nerve loss at the early stages of cardiac denervation.

## 6. PET/MRI Dual Functional Probes

Nuclear medicine imaging, and PET in particular, has a significant role in both increasing understanding of the mechanisms of cardiovascular disease and in improving patient diagnostic accuracy. However, no single modality is perfect and sufficient to provide physicians with all the necessary information. By combining new tracers with multimodality-imaging instruments that merge structural information and functional data, physicians can perform multiple functional-imaging assays simultaneously with anatomic analyses [117]. Cardiac PET/CT has been used in a variety of clinical scenarios and quantitative measurements of MBF may further enhance its diagnostic performance.

**Figure 5:**
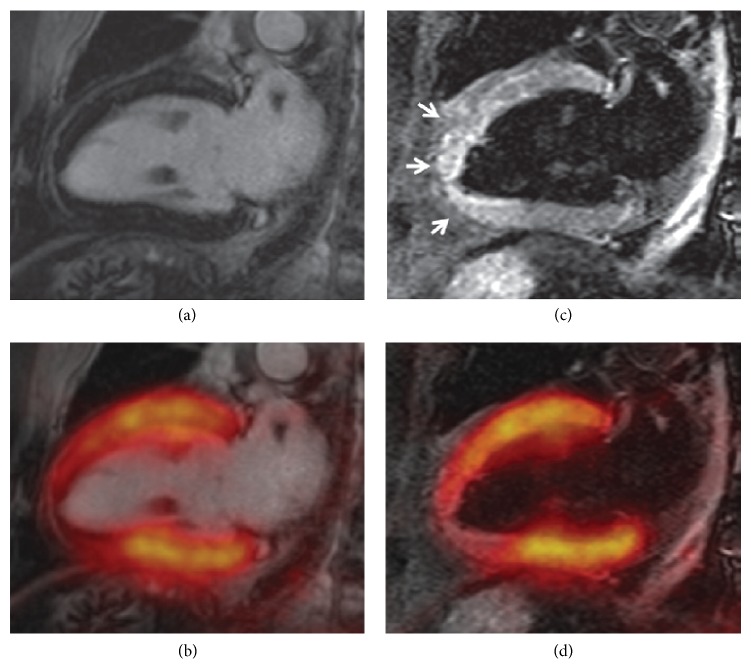
Cardiac PET/MR imaging of a 66-year-old male patient with ST-elevation myocardial infarction and acute occlusion of the left anterior descending artery. (a) Late gadolinium-enhanced (LGE) image shows no infarction zone. (b) Fused LGE and PET images show that tracer uptake was reduced. (c) MR image shows myocardial edema (arrows) that corresponded well with the area of reduced tracer uptake on (d) (from [118] with permission).

The combination of PET and MRI is another widely studied multimodality imaging technique, potentially providing opportunity for improved understanding of the mechanism and diagnosis of cardiovascular diseases. Nensa et al. [118] have demonstrated the feasibility of hybrid cardiac imaging with an integrated whole body 3-T PET/MRI that produced high-quality cardiac MR imaging acquisitions (Figure 5). MRI could provide exact anatomical localization and volume correction for detection and quantification of molecular targets by PET, with other major advantages of  PET/MRI over PET/CT including the reduction of radiation dose. With its improved tissue characterization PET/MRI has potential for atherosclerotic plaque imaging and assessment of myocardial viability/hibernation. For example, PET/MRI opens opportunities to combine anatomy (plaque burden) with haemodynamic consequences (ischaemia) of CAD [119].

Although clinical applications of such technology are still under debate, a few multifunctional probes for PET/MRI systems have already been developed for future diagnostic applications. Jerrett et al. [120] have developed dual-mode PET/MRI probes targeted to vascular inflammation, such as atherosclerosis. The probe was synthesized by coordination of the ^64^Cu to the chelating bifunctional ligand* S*-2-(4-isothiocyanatobenzyl)-1,4,7,10-tetraazacyclododecane-1,4,7,10-tetraacetic acid (*p*-SCN-Bz-DOTA) and then conjugated to the dextran sulfate coated iron oxide nanoparticle. In addition, Lee et al. [121] recently developed an iron oxide nanoparticle-based tracer for PET/MR imaging of integrin *α*_*v*_ *β*_3_ expression. Poly(aspartic acid)-coated iron oxide nanoparticles (PASP-IO) were synthesized, and the surface amino groups were coupled to cyclic RGD peptides and DOTA chelators for integrin *α*_*v*_ *β*_3_ targeting and PET imaging (after labeling with ^64^Cu), respectively. The success of this approach may allow for accurate early diagnosis of angiogenesis as well as providing molecular information specific to the disease of interest.

The future of PET/MRI is likely to benefit from the use of dual-modality PET/MRI imaging probes. However, the problem of differences in sensitivities between the two modalities remains to be solved. PET is a highly sensitive imaging modality that requires only a trace amount of probes (nanograms), whereas MRI requires a relatively high amount of contrast agent (micrograms to milligrams) [4]. Thus, the detection sensitivities for different imaging modalities should be further considered and optimized. With the improvement in PET/MRI fusion and the development of novel MRI systems with much improved sensitivity, dual-modality PET-MRI imaging agents will surely shed new light on molecular imaging of cardiovascular disease.

## 7. Conclusion

Boosted by advances in molecular biology, biotechnology, and chemistry imaging techniques, cardiovascular molecular imaging has grown rapidly in the last decade. The commonly used tracers for the noninvasive imaging of cardiovascular disease are becoming important in the paradigm shift from traditional to future imaging technologies, offering unique opportunities for improved understanding of cardiovascular disease pathogenesis and progression, and ultimately helping to optimize therapeutic interventions in patients suffering from cardiovascular disease. The development of new PET tracers that possess imaging, targeting, and therapeutic functions will remain key elements of future research in the field of cardiac PET, which will be a useful tool to monitoring new therapeutic strategies in the battle against cardiovascular disease.

## Figures and Tables

**Table 1:** Commonly used positron emitting radionuclides.

Nuclide	Physical half-life (min)	Maximum energy (MeV)	Tissue positron range (mm)^a^	Radionuclide production
^**18**^ **F**	110	0.64	0.54	Cyclotron
^**11**^ **C**	20.3	0.97	0.96	Cyclotron
^**13**^ **N**	10	1.20	1.26	Cyclotron
^**15**^ **O**	2	1.74	1.87	Cyclotron
^**82**^ **Rb**	1.27	3.38	4.10	Generator
^**99m**^ **Tc**	293.3	3.23	N/A	Cyclotron
^**68**^ **Ga**	67.7	1.90	2.12	Generator
^**64**^ **Cu**	761.4	0.653	N/A	Cyclotron

^a^Calculated for soft tissue using the full width at 20% of the maximum amplitude (FW20M) method [28].
